# Does donor-recipient body mass index ratio influence heart transplantation outcomes??

**DOI:** 10.1186/s12872-025-05147-z

**Published:** 2025-09-22

**Authors:** Matiullah Masroor, Chen Jiang, Yixuan Wang, Nianguo Dong

**Affiliations:** 1 Department of Cardiovascular Surgery, Union Hospital, Tongji Medical College, Huazhong University of Science and Technology, Wuhan, 430022 China; 2 Department of Cardiothoracic and Vascular Surgery, Amiri Medical Complex, Kabul, Afghanistan

**Keywords:** Donor-recipient BMI ratio, Heart transplantation, Recipient BMI, Donor BMI, Survival

## Abstract

**Background:**

Obesity is a common risk factor for heart failure, and heart transplantation (HTx) is the treatment of choice for end-stage heart failure. Due to the limited availability of the donor’s heart, efforts are made to increase the donor pool on one hand and to find predictors that can impact HTx outcomes on the other hand. These predictors can help improve donor organ allocation and HTx outcomes. This study aims to investigate the impact of the donor-recipient BMI ratio on follow-up mortality and other outcomes after HTx.

**Methods:**

From 2012 to 2021, 821 patients underwent HTx in our centre. Patients under 18 years, re-transplantation, multiorgan transplantation, and missing recipient and donor BMI data were excluded. The final sample size of 653 patients was divided into three quartile categories based on the donor-recipient (D-R) BMI ratio. D-R BMI ratio < 0.86 (*n* = 156), D-R BMI ratio 0.86–1.12 (*n* = 338), and D-R BMI ratio > 1.12 (*n* = 159). Analysis of variance and chi-square test with post hoc test according to the types of variables were performed to find differences among the groups. Kaplan-Meier survival analysis was used to evaluate survival, and the difference between the curves was checked with the log-rank test. Cox regression analysis was used to adjust for confounders and find independent predictors of mortality.

**Results:**

Some preoperative variables were statistically different between the groups. The D-R BMI ratio did not impact follow-up mortality after adjustment for confounders. The 7-year survival in D-R BMI ratios < 0.86, 0.86–1.12, and > 1.12 was 73%, 68%, and 70% respectively (*p* = 0.532). There was no significant difference in other postoperative outcomes, including ICU stay, systemic complications, and mechanical circulatory support use, between the groups based on unadjusted analysis.

**Conclusion:**

The donor-recipient BMI ratio had no significant impact on post-transplantation mortality. Postoperative outcomes other than survival were also comparable between the groups. These outcomes were observed in a specific D-R BMI ratio range (0.77–1.26), and extreme BMI ratios may have different results. The results of this study support the liberal use of donor and recipient BMI during patient matching, which can decrease the likelihood of potential donor non-use and subsequently increase the donor pool.

## Background

Multiple risk factors play a role in the development of heart failure, and obesity is one of those factors [1–5]. For every 1 unit increase in BMI, the incidence of heart failure increases by 5% in males and 7% in females [1]. Heart transplantation (HTx) is considered the established treatment option for end-stage heart failure [6, 7].

One of the biggest challenges in the face of HTx is the donor shortage. Many available potential allografts may be discarded because of the strict donor selection criteria [8]. The increasing use of the extended criteria donor’s hearts, such as donors of older age, donor with death from drug overdose, heart procurement from patients after circulatory deaths, use of the donors’ hearts supported with extracorporeal circulatory support, HCV, and COVID-19 positive patients, increase radius of the fly zone, and the use of repaired heart is one attempt to improve the donor pool [9]. These nontraditional donor hearts have increased the donor pool and provided hearts for many recipients, but the demand for donor hearts still outpaces the heart supply.

Therefore, researchers, while finding ways to improve the donor pool, also try to find the parameters that can positively or negatively affect the outcomes of HTx. Investigating and finding new predictors can improve donor heart utilisation and improve HTx outcomes. Several donor and recipient variables have been linked to the prognosis of HTx. Donor characteristics such as age, ischemic time, history of diabetes, left-ventricular hypertrophy, and left ventricular dysfunction increase the recipient’s risk of complications and death after HTx [8, 10–13]. Recipient characteristics such as older age, diabetes, kidney dysfunction, BMI, re-operation, and diagnosis of ischemic cardiomyopathy also affect survival and increase complications after HTx [13–16].

Additionally, donor-recipient matching is another crucial aspect that can influence HTx outcomes. Multiple parameters are used for donor-recipient matching, such as ABO blood group type [17], HLA compatibility [18], cytomegalovirus serostatus [19], ascending aortic diameter ratio [20], gender matching [21], and size matching [13]. The BMI ratio has also been used as part of size-matching metrics in some studies. The donor-recipient BMI ratio is a topic of interest and clinical importance, with very little research available. This study is mainly focused on the effect of the donor-recipient BMI ratio on HTx outcomes rather than the BMI ratio as a size matching metric.

## Methods

### Study population

**Fig. 1:**
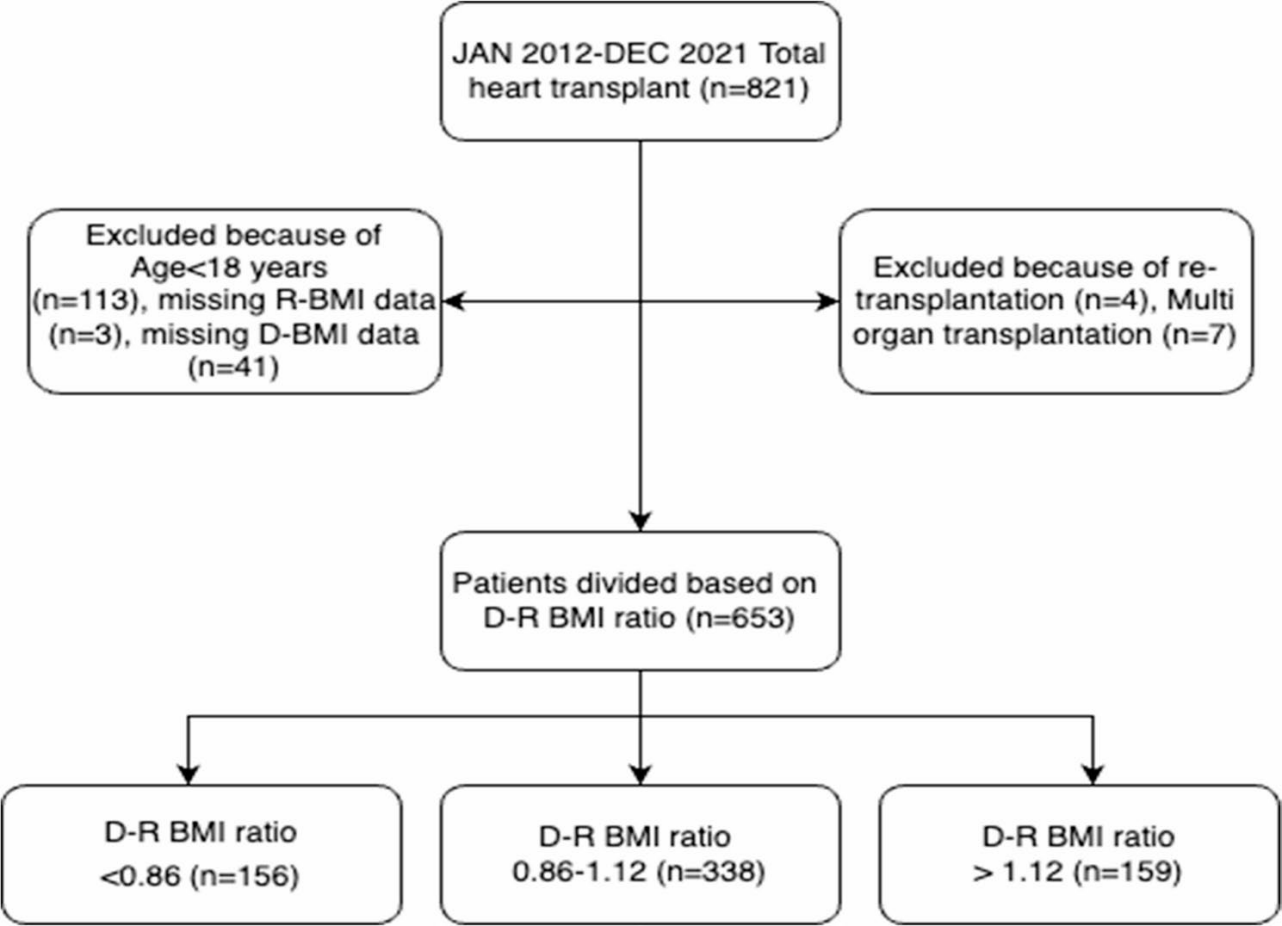
The flow chart of the study design

Between January 2012 and December 2021, 821 HTx surgeries were performed at our institution by the same surgical team. However, after excluding patients under the age of 18 years (*n* = 113), patients with missing donor BMI data (*n* = 41), recipient BMI data (*n* = 3), re-transplantation (*n* = 4), and multiorgan transplantation (*n* = 7), a final sample of 653 patients was included in the study. Both the donor and recipient BMIs may affect post-transplantation outcomes. Therefore, instead of stratifying patients based on donor or recipient BMI, we used the donor-recipient (D-R) BMI ratio (donor BMI/recipient BMI), which may predict the outcomes better than the individual donor or recipient BMI. The groups were created based on the D-R BMI ratio quartile, D-R BMI ratio less than the 25th percentile, D-R BMI ratio 25th −75th percentile, and D-R BMI ratio greater than the 75th percentile. The three D-R BMI ratio groups were: D-R BMI ratio < 0.86 (*n* = 156), D-R BMI ratio 0.86–1.12 (*n* = 338), and D-R BMI ratio > 1.12 (*n* = 159). The study design is presented in Fig. 1.

### Data collection and patients’ follow-up

The data were collected from patient records, follow-up hospital visits, and telephone contact with patients or relatives. The data includes demographic information, preoperative clinical assessments, intraoperative details, and postoperative outcomes. The follow-up was done consistently based on hospital protocols.

### Donor heart procurement and preservation method

The donor heart was arrested with 1000 mL of 8 °C HTK solution. The pulmonary veins, superior and inferior vena cava, pulmonary artery, and aorta were sequentially resected while maintaining the cardioplegia pressure at 50–70 mmHg. After removal, the donor heart was placed in a 3-layer sterile plastic bag, perfused with 1000–2000 mL of 8 °C HTK solution over 8 to 12 min. The heart was submerged in the cold HTK solution and was transported in a special box surrounded by ice cubes using public transport. During the preparation and trimming of the donor heart in the operating room, 500 mL of HTK solution was perfused through the aortic root. The left upper and the right lower pulmonary veins were marked, and the LA was opened.

### Surgical techniques

According to the donor heart arrival schedule to the centre, the recipient was shifted to the OR and anaesthetised. After the usual prep and drape, the chest was opened through median sternotomy. CPB was established through ascending aorta arterial and bicaval venous cannulation. As soon as the donor heart arrived in the OR, the leading surgeon started explanting the recipient heart after applying an aortic cross-clamp. The aorta and pulmonary artery were transected, and some parts of the LA, SVC, and IVC were preserved. At the same time, another surgeon trimmed and prepared the donor heart as discussed previously.

**Fig. 2:**
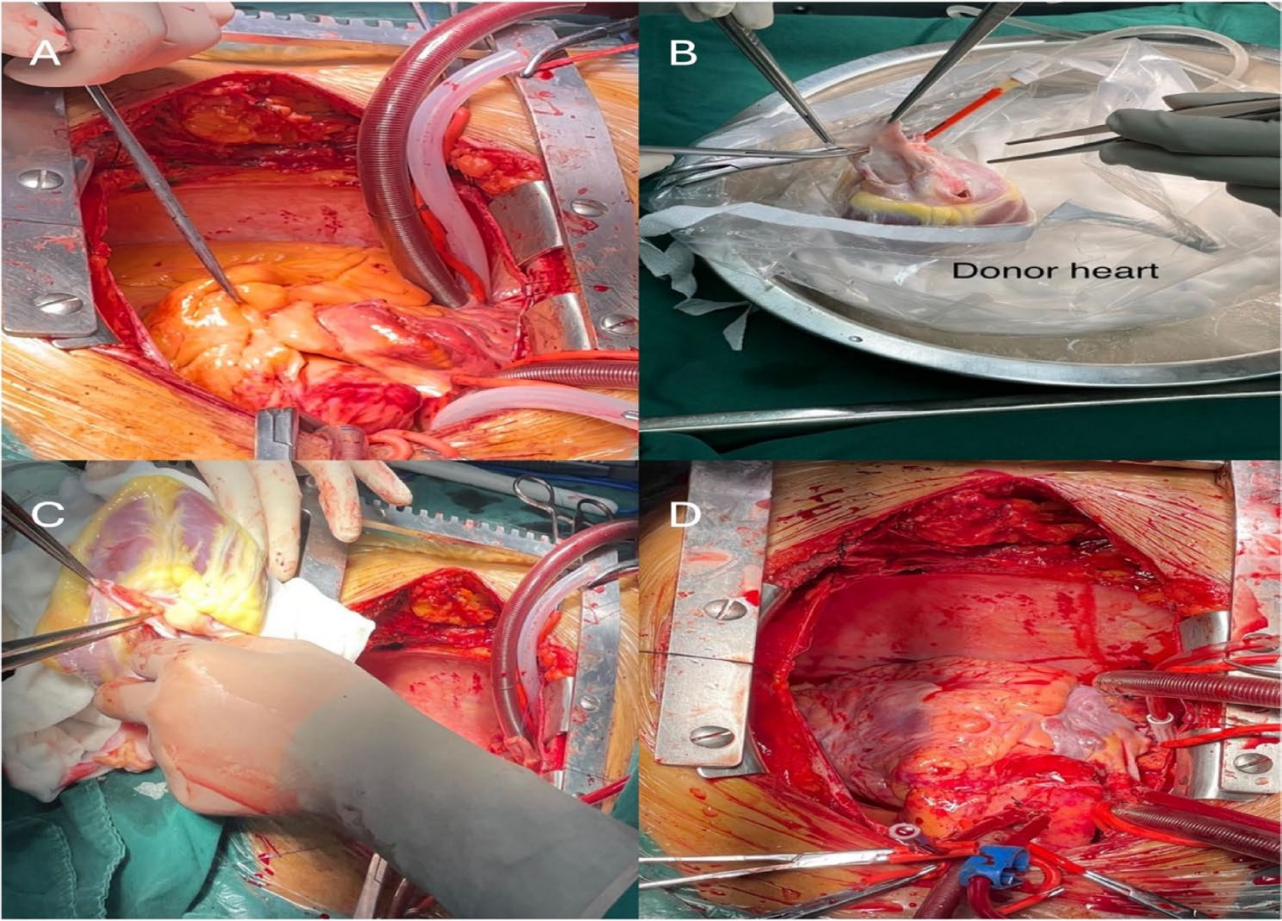
The surgical procedure of Heart transplantation. (A) Recipient’s heart before explantation (B) Donor heart during preparation and trimming. (C) Donor heart just before transplantation (D) Donor’s heart in the recipient’s chest after all the anastomoses are completed

The implantation of the donor heart started from the anastomosis of the donor’s LA to the preserved portion of the recipient’s LA, followed by the anastomosis of the ascending aorta. As soon as these two anastomoses were completed, the heart was deaired and the X-clamp was opened. The anastomoses of the IVC, SVC, and the pulmonary artery were performed on a beating heart. The HTx procedure is shown in Fig. 2. The donor heart was supported with 1/3rd of the total cold ischemic time on CPB after X-clamp opening, and before attempting to wean off the CPB. Hemostasis was secured as usual, the chest was closed layer by layer, and the patient was shifted to the ICU.

### Immunosuppression protocols

For the induction of immunotherapy, Basiliximab 20 mg and methylprednisolone 20 mg/kg were administered intravenously just before the surgery. The second dose of Basiliximab was administered intravenously on the 4th postoperative day. A standard triple-drug immunosuppression regimen, including cyclosporine A/tacrolimus, mycophenolate mofetil, and prednisone, followed this. In case of acute cellular rejection exceeding grade 2R according to the ISHLT criteria, 500 mg of methylprednisolone was administered for three days, and the doses of immunosuppressive drugs were increased.

### Statistical analysis

Statistical analysis was conducted using SPSS version 29.0.1.0. Continuous variables were presented as means and standard deviations. The means of continuous variables were compared by analysis of variance (ANOVA) to find the difference between the groups. Levien’s test of homogeneity of variance was performed for data homogeneity. The ANOVA results were used when the data conformed to the homogeneity, while the Welch robust test results were used when the data were not homogeneous. Hochberg’s GT2 test was used for post hoc analysis when data were homogenous, and Games-Howell’s test was used when homogeneity of variance was not achieved. The categorical variables were presented as frequencies and percentages. The chi-square test was used for categorical variables to compare different groups, and a pairwise chi-square test for the post hoc analysis. The Bonferroni correction was applied by dividing the alpha value of 0.05 by the number of comparisons performed to achieve the new significant alpha value. Survival rates were estimated using the Kaplan-Meier method. The Log-Rank test was used to compare different groups. An alpha value less than 0.05 was considered statistically significant. The univariable and multivariable Cox regression analysis was used to evaluate the effect of different D-R BMI ratio groups and find independent predictors for follow-up mortality after adjustment for confounders.

## Results

### Baseline patient characteristics

The three groups were created based on the D-R BMI ratio; therefore, the donor BMI, recipient BMI, and D-R BMI ratios were significantly different between the groups. After comparison of the groups, the recipient gender, diagnosis categories, donor age, recipient history of cardiac surgery, and diabetes mellitus were statistically significantly different between the groups. There was no difference in other preoperative clinical or intraoperative variables such as comorbidities, cross-clamp time, CPB time, total surgery time, and the use of preoperative mechanical circulatory support in the three groups, as presented in Table 1.

**Table 1:** Comparison of demographics, baseline clinical characteristics, and intra-operative variables of the donor-recipient BMI ratio groups

Variables	D-*R* BMI ratio < 0.86 (*n* = 156)	D-*R* BMI ratio 0.86–1.12 (*n* = 338)	D-*R* BMI ratio > 1.12 (*n* = 159)	*p*-value
Recipient age (years)	47.4±12.1	48.5±11.9	45.5±13.7	0.058
Recipient gender (male) (%)	134(85.9)	272(80.5)	110(69.2)	< 0.001
Recipient BMI (kg/m^2^)	27.1±3.6	22.8±2.5	19.5±2.3	< 0.001
Diagnosis n, (%)				0.018
NICM	103 (66.0)	204(60.4)	104 (65.4)	
ICM	33(21.2)	85(25.1)	21(13.2)	
VHD	12(7.7)	31 (9.2)	23(14.5)	
CHD	5(3.2)	9(2.7)	10(6.3)	
Others	3(1.9)	9(2.7)	1(0.6)	
Ischemic time (min)	307±122	329±117	316±101	0.147
Donor age (years)	32.8±12.0	36.2 ±11.0	38.2±10.4	< 0.001
Donor gender (male) (%)	135 (91.8)	289 (87.6)	132 (84.6)	0.154
Donor-recipient-gender n, (%)				0.002
Male-Male	120(81.6)	234(70.9)	96(61.5)	
Male-Female	15(10.2)	55(16.7)	36(23.1)	
Female-Male	6(4.1)	30(9.1)	11(7.1)	
Female-Female	6(4.1)	11(3.3)	13(8.3)	
Donor BMI (kg/m^2^)	20.7±2.5	22.3±2.2	24.4±3.6	< 0.001
D-R BMI ratio	0.77±0.07	0.98±0.07	1.26±0.13	< 0.001
Hx of cardiac surgery (%)	25(24.3)	63(28.9)	46(41.1)	0.019
Hx of diabetes (%)	32(32.3)	40(20.2)	10(11.5)	0.002
Hx of CKD (%)	9(7.6)	15(5.7)	5(3.9)	0.456
Hx of CLD (%)	6(5.0)	19(7.2)	9(7.0)	0.720
HX of PVD (%)	5(4.2)	6(2.3)	5(3.9)	0.528
HX of IABP (%)	2(1.7)	7(2.6)	1(0.8)	0.436
Hx of ECMO (%)	4(3.4)	4(1.5)	2(1.5)	0.443
Preop ventilation (%)	5(4.2)	10(3.8)	3(2.3)	0.677
Preop Ejection fraction (%)	27.4±9.8	28.4±11.9	27.3±11.6	0.505
CPB time (min)	113±33	119±66	120±41	0.374
Cross clamp time (min)	33±8	32±10	34±12	0.270
Surgery time (min)	263±99	273±96	369±80	0.642
Cause of donor death n, (%)				0.256
Traumatic brain injury	94(64.4)	197(59.9)	85(54.8)	
Cerebrovascular disease	35(24.0)	104(31.6)	59(38.1)	
Brain tumour	7(4.8)	11(3.3)	4(2.6)	
Anoxic brain death	8(5.5)	12(3.6)	7(4.5)	
Else	2(1.4)	5(1.5)	0(0.0)	

*BMI*, Body mass index, *ICM*, Ischemic cardiomyopathy, *NICM*, Non-ischemic cardiomyopathy, *VHD*, Valvular heart disease, *CHD*, Congenital heart disease, *Hx*, history, *CKD*, Chronic kidney disease, *CLD*, Chronic liver disease, *PVD*, Peripheral vascular disease, *IABP*, Intra-aortic balloon pump; *ECMO*, Extracorporeal membrane oxygenation, *LVAD*, Left ventricular assist device, *CPB*, Cardiopulmonary bypass, *TBI*, Traumatic brain injury

In the post hoc analysis for significant variables, we compared all the groups with each other. The donors of the D-R BMI ratio < 0.086 group were statistically significantly younger compared to the other two groups. Male recipients were significantly less in the D–R BMI ratio > 1.12 group compared to the other two groups, while the ICM patients were significantly less in this category compared to the D–R BMI ratio 0.86–1.12 group. Patients with a history of cardiac surgery were significantly less, and diabetics were significantly more in the D–R BMI < 0.86 group compared to the D-R BMI > 1.12 group. The probability values of the comparison of the different groups are given in Table 2.

**Table 2:** Post hoc analysis of the significant continuous and categorical variables and their probability values

Variables	D–*R* BMI ratio < 0.86		D–*R* BMI ratio 0.86–1.12
	D–*R* BMI ratio 0.86–1.12	D–*R* BMI ratio > 1.12	D–*R* BMI ratio > 1.12
Donor age	0.007	< 0.001	0.114
Recipient gender (male)	0.143	0.0004	0.005
Diagnosis			
NICM	0.227	0.908	0.279
ICM	0.333	0.061	0.002
VHD	0.588	0.056	0.077
CHD	0.763	0.199	0.049
Else	0.620	0.305	0.132
History of cardiac surgery	0.386	0.009	0.026
History of diabetes	0.022	0.0007	0.075

The Bonferroni-corrected significant alpha level for all categorical variables is 0.017, except for diagnosis, which is 0.003. The significant alpha level for continuous variables is 0.05

### Postoperative outcomes

While comparing the outcomes of D-R BMI ratio groups, there was absolutely no statistically significant difference in any outcomes between the groups, as presented in Table 3. All the important outcomes, such as ICU stay, acute rejection, systemic complications, mechanical circulatory support use, and total hospital stay, were comparable between the groups. These results are derived from unadjusted analysis, and the difference in baseline confounders can influence them.

**Table 3:** Comparison of postoperative outcomes of different donor-recipient BMI ratio groups

Variables	D-*R* BMI ratio < 0.86 (*n* = 156)	D-*R* BMI ratio 0.86–1.12 (*n* = 338)	D-*R* BMI ratio > 1.12 (*n* = 159)	*p*-value
Postop ventilation (hours)	67.9 ±186.4	71.9±154.6	81.2±194.3	0.780
ICU stay (days)	11±10.1	10.2±9	10.5±9.1	0.640
Acute rejection (%)	1(0.8)	5(1.9)	4(3.1)	0.441
Respiratory complication (%)	69(58.0)	163(61.7)	85(65.4)	0.486
Neurological complication (%)	8(6.8)	21(8.0)	8(6.3)	0.810
Renal complication (%)	19(16.2)	44(16.8)	26(20.6)	0.587
Septic shock (%)	2(1.9)	8(4.0)	5(5.3)	0.450
Positive blood culture (%)	14(12.5)	33(13.6)	21(17.6)	0.477
Positive sputum culture (%)	57 (49.6)	140(55.8)	65(52.4)	0.523
Postop EF (%)	64.7±7.4	65.1±6.3	65.3±6.1	0.705
Postop IABP (%)	59 (37.8)	119(35.2)	50(31.4)	0.488
Postop ECMO (%)	11(7.1)	19(5.6)	11(6.9)	0.772
Postop CRRT (%)	23(14.7)	43(13.0)	22(14.3)	0.841
Postop hospital stay (days)	37.0±20.1	38.4±20.4	38.0±20.8	0.776

### Survival analysis

**Fig. 3:**
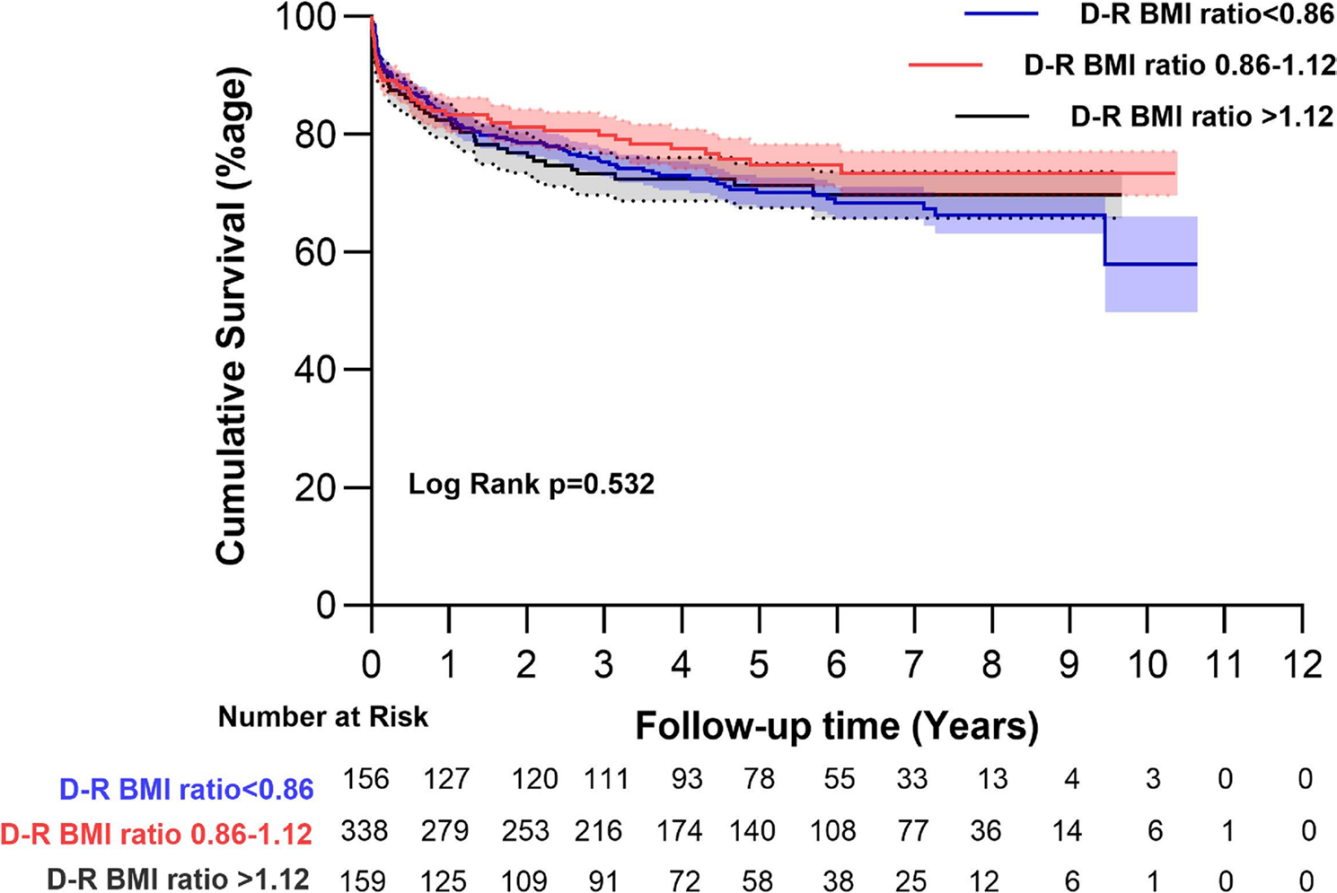
The KM survival curves of the different donor-recipient BMI ratio groups

The mean survival time was 93.5±2.2 months. The donor-recipient BMI ratio group < 0.86. One-year survival was 83.3±3.0%, 3-year survival was 79.8±3.2%, 5-year survival was 74.8±3.6%, and 7-year survival was 73.3±3.9%. The D-R BMI ratio 0.86–1.12 group, 1-year survival was 83.4±2.0%, 3-year survival was 75.2±2.4%, 5-year survival was 70.1±2.6%, and 7-year survival was 68.3±2.8%. The D-R BMI ratio > 1.12, 1-year survival was 82.3±3.0%, 3-year survival was 73.2±3.6%, 5-year survival was 71.3±3.8%, and 7-year survival was 69.7±4.0%. The follow-up survival between the groups was comparable, as the log-rank test did not find any statistical difference among the survival curves (*p* = 0.532), as shown in Fig. 3.

### Cox regression analyses

As there were some baseline differences between the D-R BMI ratio groups, to adjust for those confounders and investigate the real effect of D-R BMI ratio after adjustment, we performed univariable and multivariable Cox proportional hazard regression analyses. All the preoperative variables in Table 1 were tested through univariable analysis. The critical, significant, and non-significant variables are given in Table 4.

**Table 4:** All predictors included in the univariable Cox regression model

Variables	HR	95% CI	*p*-value
Recipient age (years)	1.030	1.016–1.043	< 0.001
D-R BMI ratio			0.534
D-R BMI 0.86–1.12	Ref	Ref	Ref
D-R BMI < 0.86	0.813	0.560-1.181	0.278
D-R BMI > 1.12	0.991	0.695-1.413	0.960
Ischemic time (min)	1.001	1000 − 1.002	0.123
Donor age (years)	1.018	1.004–1.031	0.009
D-R gender			0.161
Male-Male	Ref	Ref	Ref
Male-Female	1.489	1.023–2.168	0.038
Female-Male	1.278	0.733-2.227	0.387
Female-Female	1.420	0.744-2.711	0.288
Hx of cardiac surgery	1.294	0.889-1.884	0.179
Hx of Diabetes mellitus	1.527	0.986-2.364	0.058
Hx of CKD	2.275	1.330–3.892	0.003
HX of IABP	4.759	2.217–10.217	< 0.001
Hx of ECMO	3.116	1.271–7.639	0.013
Preop ventilation	2.427	1.187–4.964	0.015
Preop EF < 25%	0.742	0.547-1.007	0.056
Cause of donor death			0.718
TBI (%)	Ref	Ref	Ref
CVD (%)	1.179	0.851-1.634	0.323
Brain tumor	1.254	0.583 − 2.700	0.562
Anoxic brain death (%)	1.345	0.654-2.767	0.420
Else (%)	0.519	0.072-3.722	0.514

Eight variables, recipient age, D-R BMI ratio, donor age, D-R gender, history of CKD, IAPB, ECMO, and preoperative ventilation, were included in multivariable analysis based on the results of univariable analysis and clinical significance. The results of multivariable analysis showed that D-R BMI ratios < 0.86 and > 1.12 had no impact on follow-up mortality. The adjusted hazard ratio for D-R BMI ratio < 0.86 was (HR = 0.779, CI = 0.504–1.205, *p* = 0.262) while for the D-R BMI ratio > 1.12 group was (HR = 1.026, CI = 0.687–1.532, *p* = 0.902). The independent predictors for follow-up mortality were the preoperative use of IABP, history of CKD, and recipient age. The use of IABP had 5.5 times, CKD had 92%, while a one-year increase in recipient age had a 4.1% increased risk of follow-up mortality. The results of multivariable analysis are given in Table 5.

**Table 5:** Predictors included in the multivariable Cox regression model

Variables	HR	95% CI	*p*-value
Recipient age (years)	1.041	1.025–1.057	< 0.001
Donor-recipient BMI ratio			0.483
D-R BMI 0.86–1.12	Ref	Ref	Ref
D-R BMI < 0.86	0.779	0.504-1.205	0.262
D-r BMI > 1.12	1.026	0.687-1.532	0.902
Donor age (years)	1.013	0.999-1.028	0.074
Donor-recipient gender			0.997
Male-Male	Ref	Ref	Ref
Male-Female	0.960	0.610-1.512	0.860
Female-Male	0.967	0.527-1.774	0.915
Female-Female	1.028	0.487-2.167	0.943
Hx of CKD	1.927	1.123–3.306	0.017
HX of IABP	5.527	2.546–12.001	< 0.001
Hx of ECMO	1.649	0.500 − 5.440	0.411
Preop ventilation	1.026	0.305-3.447	0.967

These results show that after adjusting for baseline confounders in a multivariable Cox regression model, the D-R BMI ratio was not associated with follow-up mortality. This suggests that donor and recipient BMI can be used more liberally when matching patients for HTx. The liberal use of donor BMI will decrease the chance of donor non-use, improve organ allocation, and alleviate donor shortage.

## Discussion

The donor-recipient BMI ratio has not been studied very extensively, and the literature regarding this topic is very scarce. The available few studies about the D-R BMI ratio are focused on size matching. These studies studied the impact of the D-R BMI ratio on heart size and compared it with other size-matching metrics. A study used the UNOS registry for the adult population to evaluate different heart size matching metrics and their effect on HTx outcomes. Their study includes BMI, ideal body weight, height, predicted heart mass (PHM), body surface area (BSA), and body weight ratios. They evaluated their impact on post-transplantation survival in restrictive and non-restrictive heart failure. They found that the BMI, PHM, height, and ideal body weight ratios were significantly associated with postoperative survival, while BSA and weight ratios were not. After stratifying into restrictive and non-restrictive groups, only the BMI ratio was significantly associated with both restrictive and non-restrictive groups. They concluded that multiple size-matching metrics were associated with survival in the non-restrictive group, while only the BMI ratio as a donor-recipient size-matching metric was associated with survival in the restrictive group [22]. Another study by Lowrey et al. [23] used the UNOS registry data for pediatric HTx and challenged the traditional idea of size matching by weight. They hypothesised that matching by BMI and BSA was better associated with HTx outcomes and should be used instead of weight. They used mismatched groups by three different metrics: BMI, BSA, and weight ratio. They found that the low D-R BMI ratio was associated with one-year mortality compared to normal, while BSA and weight did not predict one-year mortality. The BMI ratio also predicted long-term survival in the non-CHD group but not in the CHD group, while the other two metrics did not. They concluded that a low D-R BMI ratio predicts worse short and long-term survival in pediatric heart transplantation and should be avoided [23]. Our results are not in line with these results. The focus of those studies was different. Those studies evaluated the efficacy of the D-R BMI ratio as a size-matching metric with other metrics. Additionally, they stratified their patients into subgroups such as restrictive vs. non-restrictive and CHD vs. non-CHD. The study of Lowrey et al. [23] was focused only on the pediatric population. We found that the D-R BMI ratio had no impact on post-transplantation survival and outcomes. Our study includes only adult patients, and we did not focus on size matching. We simply evaluated the effect of the D-R BMI ratio on outcomes, including survival. One possible reason for not observing any outcomes and survival difference among the different D–R BMI ratio groups in our study may be the very little difference in the heights of the donors and recipients and the huge difference in the weights. In other words, the difference in BMI among the different groups is mainly driven by weight. The weight-driven BMI difference may underestimate the real power of the BMI if it were achieved through different donor and recipient heights and weights. Therefore, in this situation, careful donor and recipient selection based on the donor-recipient BMI ratio may be required. The limited number of studies with differences in results warrants prospective large-sample-size studies.

Kransdorf et al. [14] analysed the ability of five size-matching metrics, PHM, BMI, weight, height, and BSA, to predict one-year mortality after HTx and found that PHM was the best indicator among all for predicting post-transplant mortality. When donors were undersized by other metrics, they were not associated with 1-year follow-up mortality. So, the result of our study is in line with their study, suggesting that the undersized group of D-R BMI ratio has no association with survival. One recent study by Zhong et al. [24] studied the impact of D-R BMI ratio on post-transplantation outcomes, but they divided patients into two groups based on D-R BMI ratio ≥1 and < 1. They concluded that the D-R BMI ratio ≥1 group has higher postoperative mortality compared to the BMI < 1 group. On multivariable analysis, the relative risk of mortality was 50% higher in the BMI ≥1 group (HR: 1.50, CI: 1.08–2.09, *p* = 0.015) [24]. We believe that the stratification of patients into a BMI of < 1 and > 1 may not give us accurate information about the patient’s condition. Both donors of BMI 45 kg/m^2^ and 25 kg/m^2^, with a recipient of BMI 22 kg/m^2^, will give us a D-R BMI ratio > 1, which is not clinically similar. We believe the better study design would be dividing patients into four or five recipient BMI categories and then further dividing them into subgroups based on the D-R BMI ratio. In our study, this is not possible because of the limited number of patients. Still, registries’ databases can perform such studies, which will give us a clear picture regarding the D-R BMI ratio’s impact on HTx outcomes.

## Limitations

The limitations of this study are its retrospective observational nature, which can not fully control the risk of confounding factors. Outcomes other than survival are not derived from adjusted data and can be influenced by baseline variable differences. The unmeasured variables, such as pulmonary vascular resistance, HLA and CMV matching, PHM data, etc., can affect the outcomes. The very low and very high D-R BMI ratios may have worse outcomes, which we can not affirm from this study, as the mean D-R BMI ratio in the lower and upper D-R BMI groups was 0.77±0.07 and 1.26±0.13, respectively. Future multicenter studies with a prospective design, complete data, and a large sample size can further clarify the impact of D-R BMI ratio on HTx outcomes.

## Conclusion

Our study found that the donor-recipient BMI ratio was not associated with post-HTx short and long-term survival after adjusting for confounders. Postoperative outcomes other than survival were comparable between the groups based on unadjusted analysis. The outcomes are observed across the D-R BMI ratio range (0.77±0.07 to 1.26±0.13), but extreme BMI ratios remain unstudied and may have different outcomes. Based on our results, we believe that the liberal use of donor and recipient BMI while matching the patients can decrease potential donor heart non-use and subsequently increase the donor pool.

## Data Availability

The dataset analysed during the current study is available from the corresponding author on reasonable request.

## Abbreviations

HTx: Heart transplantation
BMI: Body mass index
D-R: Donor-recipient
HLA: Human leukocyte antigen
HTK: Histidine, tryptophan, and ketoglutarate
LA: Left atrium
SVC: Superior vena cava
IVC: Inferior vena cava
